# Mutual learning globally

**DOI:** 10.3402/ejpt.v4i0.21241

**Published:** 2013-06-06

**Authors:** Ulrich Schnyder

**Affiliations:** Department of Psychiatry and Psychotherapy, University Hospital Zurich, Switzerland

**Keywords:** trauma, ESTSS, ISTSS, Global Collaboration, cultural sensitivity, mutual learning

## Abstract

This article is about the development of the trauma field over the last 20 years from an organizational perspective, and about trauma from a global, culture-sensitive perspective. My professional career is very closely linked to the development of the European Society for Traumatic Stress Studies (ESTSS) in the 1990s. Later on, I was fortunate enough to witness, and contribute to, the International Society for Traumatic Stress Studies’ (ISTSS) increasing focus on trauma as a global issue. I am trying to demonstrate how important the ESTSS and the ISTSS have been for me, how serving these societies has shaped my thinking, both as a clinician and a researcher, and how much I learned from these experiences.

I became what I am today at the age of twelve, on a frigid overcast day in the winter of 1975. I remember the precise moment, crouching behind a crumbling mud wall, peeking into the alley near the frozen creek. That was a long time ago, but it's wrong what they say about the past, I've learned, about how you can bury it. Because the past claws its way out. Looking back now, I realize I have been peeking into that deserted alley for the last twenty-six years.

I frequently use these very first few sentences of Khaled Hosseini's book The Kite Runner (Hosseini, 2003) as an introductory citation in my lectures on trauma and posttraumatic stress disorder (PTSD), as an example from the literature on the long-term repercussions of traumatic exposure on people's lives. Paraphrasing Hosseini, I might say with regard to my professional career, “I became what I am today on a morning in June, 1993, when I ran into Lars Weisæth who took me by the hand and dragged me into a meeting room at a conference Hotel in Bergen, Norway.” I will get back to that later.

This article is about the development of the trauma field over the last 20 years from an organizational perspective, and about trauma from a global, culture-sensitive perspective. I am neither a historian nor am I one of the pioneers who shaped the field of modern psychotraumatology from its inception in the 1980s. However, my professional career is very closely linked to the development of the European Society for Traumatic Stress Studies (ESTSS) in the 1990s. Later on, I was fortunate enough to witness (and contribute to) the International Society for Traumatic Stress Studies’ (ISTSS) increasing focus on trauma as a global issue. It goes without saying that, due to my personal involvement, my account will be strongly biased. This will not be an objectively accurate report. Rather, I will try to demonstrate how important the ESTSS and the ISTSS have been for me, how serving these societies has shaped my thinking, both as a clinician and a researcher, and how much I learned from these experiences.

## The European Society for Traumatic Stress Studies (1993–2001)

I am a late bloomer, in many respects. I had studied medicine and intended to become a general practitioner. After 5 years of residencies in pathology, gynecology and obstetrics, surgery, and internal medicine, I changed my mind and started training as a psychiatrist and psychotherapist. I got interested in emergency psychiatry and crisis intervention. This was where I first met Berthold Gersons who was to become my predecessor on the ISTSS Board of Directors, and my successor on the ESTSS Board of Directors, many years later. I did a few studies (Schnyder, 1995), published a book (Schnyder, 1993), and tried, unsuccessfully, to join related professional organizations, such as the International Association for Emergency Psychiatry. I was unhappy. I felt disconnected, left out, unwelcome in the academic arena. I was also very ambivalent: Did I want to be a clinician, or an academic researcher, or both? I did not quite know.

In 1990, I had attended my first conference on traumatic stress, the second European Conference on Traumatic Stress (ECOTS), Noordwijkerhout, The Netherlands. I was impressed by the plenary lectures of Lars Weisæth, Lenore Terr, Bessel van der Kolk, and Jack Lindy. I did not present anything myself, I just tried to absorb what the “pioneers” were discussing. At the first World Congress on Traumatic Stress in Amsterdam in 1992, I listened to Beverly Raphael, Tom Lundin, Charlie Marmar, Bob Pynoos, Rachel Yehuda, Arik Shalev, Roger Pitman, Charles Figley, Judith Herman, Dean Kilpatrick, and many others. The third ECOTS, held on June 6–10, 1993, in Bergen, Norway, was my third trauma conference. I presented my first paper, some preliminary data on knowledge about and clinical experiences with trauma-related disorders among Swiss psychiatrists and general practitioners (Schnyder, 1996). I was still a newcomer to the field. The only person I knew a bit was Lars Weisæth whom I had invited 2 years previously to give a lecture on his work with the Scandinavian Star disaster of 1990 at a symposium on crisis intervention in Bern, Switzerland. So, when Lars grabbed my hand saying “come with me”, and dragged me into that meeting room on June 8, the second last day of the conference, I readily complied.

We arrived late. Some 10–15 people were sitting around a long table. I did not know anybody. Lars introduced me to what turned out to be the pioneers of European Psychotraumatology: Wolter de Loos, Stuart Turner, Roderick Ørner, Atle Dyregrov, Louis Croq, Tom Lundin, Wybrand op den Velde, and others whose names I do not recall. I understood this was the meeting that launched the ESTSS. I was excited and deeply impressed. Wolter de Loos was appointed President, Stuart Turner Vice-President and Petra Aarts Secretary. There was lively, if not heated discussion among the members of the founding board about each and every agenda item. Finally, a Treasurer had to be found, and, naturally, nobody volunteered. All of a sudden, somebody said “… hey, we have a Swiss guy here! He certainly knows how to take care of the ESTSS’ finances!” I tried to decline, but the group did not give me a chance. I was appointed Treasurer of ESTSS.

I had no idea whatsoever what a Treasurer's tasks were. It was not so difficult in the beginning, though. Before the end of the conference, we had collected about 50 addresses of colleagues who wanted to join as members, plus, if I recall correctly, a membership fee of 100 Norwegian Krones from each of them. I started building a database. I got great support from Arthur Blank junior who was ISTSS Treasurer at the time. Petra opened a bank account. The Executive started circulating draft by-laws. The ESTSS was born!

The ESTSS’ infant and early childhood development, however, was complicated. There were tensions from the very beginning, both within the ESTSS, and between ESTSS and ISTSS. There were financial problems. There was, to put it diplomatically, a certain amateurism with regard to our organizational and administrative skills. Also, to our disappointment, the society did not grow as fast as we had hoped and expected. On the other hand, there was a great deal of enthusiasm, a pioneering spirit, a willingness to make a difference and help developing the emerging field of psychotraumatology. We felt that while being a bit behind the United States and the ISTSS organizationally, European professionals should be able to make unique contributions to both the theory and practice of trauma work. European cultural background and our specific experiences with large-scale trauma in recent history, particularly WW II and the holocaust, were emphasized at the fourth ECOTS. The conference was held from May 7 to 11, 1995, in Paris, France. On May 8, Europe commemorated the 50th anniversary of the WW II armistice, so the fourth ECOTS took place at a time when there was full media coverage on the holocaust. Louis Croq, the local conference organizer, not only emphasized European scientific traditions; he also spoiled participants with a classical concert in a church, and French cuisine on a bâteau-mouche on the river Seine!

I ended up serving as ESTSS Treasurer for 6 years, under the presidencies of Wolter de Loos (The Netherlands, 1993–1995), Stuart Turner (UK, 1995–1997), and Roderick Ørner (UK, 1997–1999). Frankly speaking, my enthusiasm with regard to the Treasurer job was limited. What I did enjoy immensely, however, was sitting on the Board, and serving as a member of the Executive. The launch of the ESTSS really launched my career as an academic. As a member of the ESTSS Executive, I got in touch with the most creative thinkers, the pioneers of the emerging field of traumatic stress research. All of a sudden, I did feel, and actually was, connected and welcome.

I got first-hand information about the latest developments in the field of traumatic stress research. I learned about novel therapeutic approaches. I participated in heated debates about controversial issues, such as Critical Incidence Stress Debriefing, the False Memory Syndrome, or Eye Movement Desensitization and Reprocessing. The controversy about the usefulness of Critical Incident Stress Debriefing, by the way, inspired the ESTSS Board to launch a book series on European Perspectives on Psychotraumatology with Oxford University Press. Roderick Ørner and myself committed to edit the first volume, Reconstructing Early Intervention after Trauma, which was published in 2003 (Ørner & Schnyder, 2003). We collected the evidence available at the time and came forward with a number of suggestions as to how early interventions after trauma might be “reconstructed”.

And, of course, I learned a lot about how to establish and successfully run a professional membership society, plus about the many pitfalls you can stumble into, and mistakes you can make along the way. This is maybe my most important conclusion about serving on the Board of Directors of a professional organization: I learned a lot. On top of that, I experienced something that may not be equally generalizable: I found my professional home. I started belonging to this exciting and enthusiastic group of colleagues. I wanted to become one of those people who really seemed to care about what they did, who had a vision and a mission around the issue of traumatic stress, or psychotraumatology as European professionals started to call the developing field. I started focusing my research on trauma (Schnyder, Büchi, Mörgeli, Sensky, & Klaghofer, 1999), and studied evidence-based psychotherapies for PTSD. According to George Engel, I wanted to become a “scientific physician”, rather than a “physician–scientist” (Engel, 1987).

In 1999, I was elected President of the ESTSS and served in that role until 2001. Bas Schreuder took over as Treasurer, and Karen Sadlier was appointed Secretary. While being the culmination of my “ESTSS career”, this was also a time of transition already: As ESTSS President, I attended the Board meetings of the ISTSS as an ex-officio, non-voting member. During that time, I learned my first lessons regarding the similarities and differences of ESTSS and ISTSS. The level of enthusiasm and commitment seemed similar. However, ISTSS was much stronger at that time already, not only regarding their financial power, but the development of their organizational structure, policies, and procedures as well. Thus, serving on the ISTSS Board was, and probably still is, different from the ESTSS. The ISTSS is much larger than the ESTSS I knew from the 1990s. Although, following its organizational development into a federation, the ESTSS now represents a similar number of trauma professionals, the ISTSS is stronger in terms of management, with several permanent staff members running the society, and an annual budget of currently over US$ 1.4 million.

## The International Society for Traumatic Stress Studies ISTSS (1999–2013)

As mentioned above, during my presidential term with ESTSS (1999–2001), I was an ex-officio member of the Board of Directors of the ISTSS. A year later, in 2002, I was elected to the ISTSS Board as a regular Board member. Once again, contrary to my early experiences with the Emergency Psychiatry community, I felt welcome. Also, in contrast to how ISTSS was (and still seems to be) perceived from the outside, about one-third of the Board members were from outside the United States. Thus, in spite of over 80% of individual ISTSS members being North America based, its Board of Directors showed a fairly strong international representation. I will get back to this a little later.

I soon got quite enthusiastically engaged in the ISTSS. I served on several committees and task forces, including the Finance Committee (which, in my view, is one of the best ways to get to know the nuts and bolts, and learn about the functioning of an organization), International Structure and Affiliations Committee, Organizational Liaisons Committee, Policies and Procedures Task Force, Awards Committee, Nominations Committee, Annual Meeting Committee, and the Scientific Program Committee for several ISTSS Annual Meetings. I also served on the Executive Committee of ISTSS as Vice-President for 2 years: in 2003/2004, with Paula Schnurr as President; and in 2007/2008, with Stuart Turner as President.

At ISTSS, board members are usually elected for a 3-year term. You can run for a second term, however, after a maximum of 6 years, you are not eligible for re-election for at least 1 year unless you are elected President-elect. This was exactly what happened to me, so I served a third 3-year term, as President-elect, President, and Past President.

This last 3-year term was probably the most interesting, intense, both challenging and rewarding time of my engagement with ISTSS. Once again, I learned a lot: about the principles of leadership in a volunteer organization; about the interaction between elected officers and employed staff; and, most importantly, about the importance of cultural sensitivity (Tseng & Streltzer, 2001) when trying to move ahead with a group of highly committed professionals, many of whom come from totally different cultural backgrounds. I learned about institutional resistance to change. And I experienced more than once that if you succeed in listening to the people you work with, and really try to understand and respect their viewpoints, something that I would like to call “mutual learning” may occur. These are the moments I truly love, the moments that make it worthwhile to engage in the complexities of intercultural exchange.

In November 2011, I stepped down from the ISTSS Board. Taking the ESTSS and ISTSS Boards together, I had served on these boards in various roles for 18 consecutive years. Needless to say it was time to resign, and make space for others.

## Trauma is a global issue

The fact that trauma is a global phenomenon is an issue that both ESTSS and ISTSS have been struggling with long before I joined the trauma field. There is increasing consensus that the most pressing challenges in our field can only be met if we understand trauma from a cultural perspective, in addition to the psychological, social, neurobiological, legal, political, etc. perspectives we use to take when looking at a specific aspect of trauma. When in 1991, the Society for Traumatic Stress Studies added an “I” for “International” to its name, thus becoming the ISTSS, leaders of the organization had recognized the global dimension of trauma. Nevertheless, ever since, around 80% of the ISTSS members are North American residents. As a consequence, the ISTSS annual meetings have always been held in North America, i.e., the United States or Canada. Why is that? Well, the answer is simple: Holding the annual meeting anywhere outside North America would inevitably attract fewer participants. With more than 50% of ISTSS’ income being generated by the annual meeting, this would create a major financial challenge to the Society. At any rate, due to the fact that most of its members are North Americans and its annual meetings are invariably held in North America, and despite an increasing number of ISTSS sponsored activities on a global level, the ISTSS was perceived by many of our colleagues worldwide as not being a “truly international” Society.

In 2010, the ISTSS, as part of its new strategic plan, explicitly recognized that traumatic stress is a global issue, and decided to seek to have a stronger global impact on trauma-related issues. As a result, the “Global Initiative” was created (Worth, 2010). A project team was appointed and mandated to develop concrete proposals for action. One of the action packages approved by the ISTSS Board of Directors is the Global Collaboration: ISTSS and its affiliate societies agreed to work alongside each other on an equal basis, to identify objectives, facilitate development, and coordinate activities of global importance. This effort began with ISTSS and its affiliate societies but is intended to encompass the broader trauma community in the future.

In November 2012, the Global Collaboration achieved agreement to focus on one global issue to start, namely childhood abuse and neglect and the latent impact of that abuse. The group decided to collect guidelines from around the world that would provide the basis for a synthesized core guideline for prevention and treatment that can be customized for specific cultural contexts. The guideline will primarily be aimed at professionals. In addition, the Collaboration will compile an information guide aimed at those affected by childhood abuse and neglect. This will raise awareness of the issue, improve the way individuals of all ages who are affected by childhood abuse and neglect are detected, supported, assessed, and treated, leading to significant improvements in health and wellbeing. Capitalizing on the latest developments in technology, the Global Collaboration aims to disseminate these guidelines using an application for mobile electronic devices that will allow for worldwide distribution and cultural customization.

For the time being, the Global Collaboration is chaired by Miranda Olff—past president of ESTSS, the current vice-president of ISTSS, and founding editor-in-chief of the *European Journal of Psychotraumatology*. I am truly delighted and grateful to see the Global Initiative coming to fruition. Trauma is a global issue. I hope and trust that the European view of psychotraumatology will impact strongly and creatively on the Global Collaboration!

## Conflict of interest and funding

The author declares no conflict of interest in the present article.
